# Comparative transcriptome analysis of unripe and ripe banana (cv. Nendran) unraveling genes involved in ripening and other related processes

**DOI:** 10.1371/journal.pone.0254709

**Published:** 2021-07-27

**Authors:** Karambir Kaur, Praveen Awasthi, Siddharth Tiwari

**Affiliations:** Department of Biotechnology, Plant Tissue Culture and Genetic Engineering Lab, National Agri-Food Biotechnology Institute (NABI), Ministry of Science and Technology (Government of India), Mohali, Punjab, India; ICAR-Indian Institute of Agricultural Biotechnology, INDIA

## Abstract

Banana is one of the most important fruit crops consumed globally owing to its high nutritional value. Previously, we demonstrated that the ripe pulp of the banana cultivar (cv.) Nendran (AAB) contained a high amount of pro-vitamin A carotenoids. However, the molecular factors involved in the ripening process in Nendran fruit are unexplored. Hence, we commenced a transcriptome study by using the Illumina HiSeq 2500 at two stages i.e. unripe and ripe fruit-pulp of Nendran. Overall, 3474 up and 4727 down-regulated genes were obtained. A large number of identified transcripts were related to genes involved in ripening, cell wall degradation and aroma formation. Gene ontology analysis highlighted differentially expressed genes that play a key role in various pathways. These pathways were mainly linked to cellular, molecular and biological processes. The present transcriptome study also reveals a crucial role of up-regulated carotenoid biosynthesis pathway genes namely, *lycopene beta cyclase* and *geranylgeranyl pyrophosphate synthase* at the ripening stage. Genes related to the ripening and other processes like aroma and flavor were highly expressed in the ripe pulp. Expression of numerous transcription factor family genes was also identified. This study lays a path towards understanding the ripening, carotenoid accumulation and other related processes in banana.

## Introduction

Banana is amongst one of the most essential staple food cultivated in both tropical and subtropical countries and consumed worldwide [1]. The banana plant is a flowering monocot belonging to the family Musaceae and mainly originated from intra- and inter-cross among *Musa acuminata* (A genome) and *Musa balbisiana* (B genome) [2]. This resulted in several genome groups viz. AA, AB, AAA, AAB, ABB, AABB, AAAB and ABBB [3]. Ripening process in banana leads to various changes in gene expression that results in changes in flavor, texture and color of these fruits [4, 5]. These irrevocable biological and physiological changes due to ripening often result in shortening of the shelf life of a banana, causing losses at its postharvest level [6] Previously, some chemical treatments were extensively employed to minimize postharvest losses but due to economical and health concerns, these were not favored [7]. Previous studies have reported that ripening in banana involves various gene families that are associated mainly with cell wall degradation and few genes have also been identified which are associated with transcription factors (TFs), signal transduction and ethylene biosynthesis [8–10]. However, the role of molecular factors related to carotenoid accumulation during the ripening process is not explored much in banana. Carotenoids play a significant role during fruit ripening and banana represent a low to moderate amount of their content. Screening of banana germplasm is found to have a significant variation in carotenoid content in their pulp tissue [11]. Banana cultivar (cv.) Nendran (AAB) is identified with high pro-vitamin A content in ripe fruit-pulp [12]. Hence, it will be alluring to study the molecular mechanism associated with ripening and pro-vitamin A accumulation in Nendran.

DNA sequencing has grown as an inescapable medium for studies related to molecular biology. The availability of the draft sequence of the banana genome (523 megabase) from *Musa acuminata* a double haploid provided imperative information for genetic improvement of the banana plant [13]. Being a quick and economical method, next-generation sequencing (NGS) tools provide high throughput transcriptome analysis with techniques like RNA-Seq [14]. In comparison to whole-genome sequencing, transcriptome profiling is favored as it is confined to study only a subset of the genome (transcribed portions of the genome) [15]. This analysis helps to understand the expression of genes in varying biological environments like in cells and tissues [15]. Further, factors related to stresses and different metabolic pathways can be well studied using genome-scale NGS based technologies. In banana (Dwarf Cavendish), transcriptome analysis has been done to understand the molecular mechanisms involved during the ripening process [16]. Transcriptome analysis of different varieties of *Musa* spp. has been performed on leaf, root, pulp, rhizome in response to fungal infection, to analyze the role of TF in ripening and to study metabolic processes under low potassium stress [17]. These analyses could further enhance our understanding of the molecular mechanism underlying biosynthesis and defense, and can also contribute to elucidate evolutionary aspects of its genes, and genomes. Databases such as Arabidopsis Next-gen sequences DBs and prediction algorithms have been used to provide information on genes associated with fruit ripening [18]. Further, transcriptome analysis of fruits like kiwi [19] blueberry [20], *Cucumis melo* [21], orange [22, 23] watermelon [24] and tomato [25] have led to identifying pathways and genes associated with fruit ripening and development. Recent developments in genomics comprising molecular markers (simple sequence repeats) have also enhanced our current knowledge in understanding various functional characteristics of the plant genome, which can help to improve banana by breeding approaches [16]. *In-silico* databases also use to harbor information of novel molecular markers that can be utilized for genetic improvement programs in banana.

The present study is commenced to get the global expression profile about the key molecular factors involved in ripening, carotenoid accumulation and other related processes in the economically and nutritionally important banana cv. Nendran. To elucidate the role of various up- and down-regulated genes in Nendran, we have generated and analysed transcriptomic data at two fruit developmental stages i.e. unripe and ripe using NGS technology hosted on the Illumina platform.

## Materials and methods

### Plant material

Banana cv. Nendran was used for experimental purposes. The fruit samples from unripe (6 weeks/w) and ripe (15 weeks/w) stages of Nendran were collected from the banana germplasm plot at National Agri-Food Biotechnology Institute (NABI), Mohali, Punjab. Sampling was performed during the summer and winter seasons. The tissues were then kept in liquid nitrogen and stored at 80°C till further use.

### RNA extraction, cDNA library preparation and illumina sequencing

Total RNA was isolated from fruit pulp using the RNA extraction kit (Sigma-Aldrich, USA). In total three biological replicates were taken for each sample and used further for the isolation of RNA. Isolated RNA was treated with DNase I kit (Ambion Thermo Scientific, USA) to eliminate DNA contamination. Total RNA was analyzed by agarose gel electrophoresis for size and integrity. The quantification of total RNA was done with a NanoQuant (Infinite 200 PRO NanoQuant, Austria). The sample for RNA sequencing was derived from the pooling of the RNA samples in two groups i.e. replicates isolated from the fruit-pulp of 6w (unripe) and 15w (ripe) stages. DNA-free RNA was used for cDNA first-strand synthesis by using revert aid first-strand cDNA synthesis kit (Thermo Scientific, USA) as per manufacturer’s protocol. Oligo dT primer was used for cDNA preparation. Consequently, the integrity of RNA used for library preparations was checked with a value of ≥ 8.5 using Bioanalyzer (Agilent, USA). The quality control (QC) passed RNA samples were then processed for library preparation. The paired-end libraries were prepared from the total RNA using Illumina TruSeq stranded mRNA library prep kit as per the instructions (Illumina Inc., USA). The generated libraries were sequenced on Illumina HiSeq 2500 platform.

### Transcriptome assembly and RNA seq analysis

Raw reads obtained from sequencing were processed to obtain high-quality reads. Moreover, all reads were trimmed by using the Trimmomatic 0.35 tool [26] to remove low-quality reads and any adapter sequences if present. The resultant high-quality reads of each sample were used for mapping on *Musa acuminata* DH-Pahang v2 on banana genome hub [27] (https://banana-genome-hub.southgreen.fr/download). The reads were mapped using STAR 2.6 [28]. Cufflinks v2.2.1 [29] program was used to assemble the STAR aligned transcripts to quantify their expression. Cufflinks, Cuffmerge and Cuffdiff were then used for further mapping and expression analysis of differentially expressed genes (between unripe and ripe samples). Cuffdiff software was also used to quantify the abundance of transcripts in the form of Fragment Per Kilobase of transcript per Million mapped reads (FPKMs). The genes were additionally categorized as differentially expressed by considering statistical significance (p<0.05, p<0.01) and false discovery rate for significant expression.

### Functional annotation of Differentially Expressed Genes (DEG) and pathways

For functional annotation of DEG and to identify putative pathways associated with them, we annotated identified DEG’s with banana genome hub, NCBI protein database and GO databases. Significant GO IDs were extracted from the banana genome hub ontology browser. The g:Profiler web server was employed for functional enrichment analysis [30]. Further, the WEGO tool [31] was used to calculate the statistical enrichment of DEGs in various pathways using FDR values of < 0.05 (threshold).

### Quantitative real-time PCR (qRT-PCR) validation

Total RNA was isolated from ripe and unripe fruit pulp samples and cDNA was prepared as discussed above. The qRT-PCR study was implemented with ABI 7500 Sequence Detector (Applied Biosystems, USA). Housekeeping gene *Actin1* (GenBank Accession No. AF246288) was used to normalize the variant expression of chosen genes [32, 33]. The primers were firstly tested for single-band amplification using conventional end-point PCR. The expression of each gene was tested in unripe and ripe conditions of Nendran fruit samples. A melting curve study was carried out using qRT-PCR. The total volume of each reaction was adjusted to 10 μl and contained 1X SYBR Green Master Mix (Applied Biosystems, USA); 5 pmol of each primer (forward and reverse); 0.5 μl cDNA template and sterile distilled H_2_O. PCR conditions followed during real-time PCR experiment were: step (1) 50°C 2 min, step (2) 95°C 10 min, step (3) (95°C 0.15 min, 60°C 1 min) x 40 cycles, followed by the thermal dissociation curve. The relative expression level was analyzed using the 2^-ΔΔCt^ method [34]. Primer details along with corresponding gene IDs are mentioned in the **S1 Table**. All the primers used in the qRT-PCR analysis were unique to each gene and were designed using Primer3 software [35]. All experiments were executed in biological triplicates and each experiment entailed three technical replicates. Statistical significance was determined by using the Student’s paired t-test.

## Results

### Transcriptome sequencing, alignment and analysis of banana fruit samples

The whole transcriptome sequencing i.e. RNA-seq (paired-end) of fruit (ripe and unripe) samples of cv. Nendran was performed using Illumina HiSeq 2500 platform. On average for each sample, 96,013,558 reads in NEN-Ripe and 107,849,342 in NEN-Unripe samples were recovered from two cDNA libraries. Approximately 89.30% of total reads have a Phred quality score > 30 (a measure of the quality of nucleobases). After exclusion of low-quality reads, 95,320,622 and 107,351,986 reads were obtained from NEN-Ripe and NEN-Unripe samples, respectively. Clean reads were then selected for aligning to the banana genome. By mapping the selected transcripts, 94% (Ripe) and 92% (Unripe) reads matched with the banana genome (**Table 1**).

**Table 1:** RNA sequencing statistics of ripe and unripe banana cv. Nendran samples.

Sample Name	Total Read Count	Read Count after rRNA removal	QC Pass %	Aligned Read Count	Aligned %	Unaligned %
NEN-Ripe	96,013,558	95,320,622	99.28	8,99,28,387	94.34	5.66
NEN-Unripe	107,849,342	107,351,986	99.54	9,91,05,193	92.32	7.68

**Fig 1:**
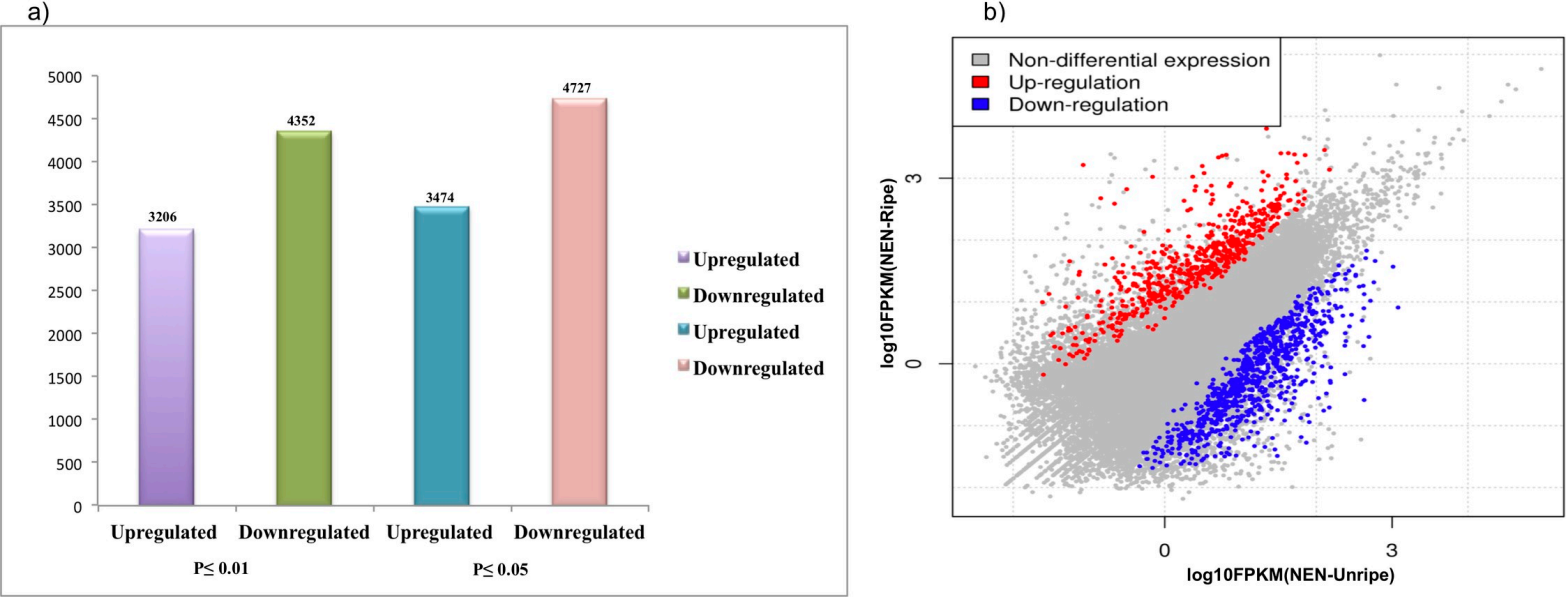
General features of Nendran banana transcriptome. a) Total number of DEGs with p-value ≤ 0.01and ≤0.05. b) Scatter plot of all differentially expressed genes. Significant genes with log fold change >2 are indicated by red color and those having log fold change <2 are indicated by blue color, while genes without significant difference are depicted by grey color.

Further, the expression is evaluated in FPKMs using the Cufflink software package [29]. Based on log2 fold change parameter and p-value ≤ 0.01, we obtained 3206 up-regulated (≥ 2 fold) and 4352 (≤ -2 fold) down-regulated genes in unripe vs. ripe samples (**Fig 1A**). Similarly, with p-value ≤ 0.05, a total of 3474 up- and 4727 down-regulated genes were obtained (**Fig 1A**). The scatter plot of the expressed genes at unripe and ripe stages of fruit-pulp is presented in **Fig 1B**. Different colors were used in scatter plot to specify up-regulated genes, down-regulated genes and genes in which expression was not affected. We evaluated unripe and ripe samples and created scatter plot of expressed genes where specific colors were used to exemplify down-regulated, up-regulated and non-regulated genes. The DEGs were examined between control (Unripe) and test (Ripe) samples using the FPKM method log2 fold change ≥ 2 as a threshold. The signifying log2 values of gene expression and screening conditions are represented in **Fig 1B**.

### Functional enrichment of differentially expressed genes

**Fig 2:**
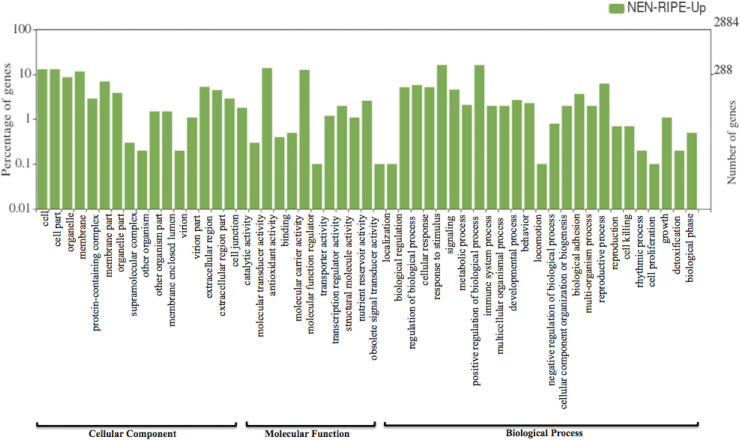
WEGO plot representing annotation and classification of differentially expressed genes in Nendran ripe fruit-pulp. X-axis (right) displaying selected GO terms of up-regulated genes and y-axis (left) displaying the percentage of genes.

**Fig 3:**
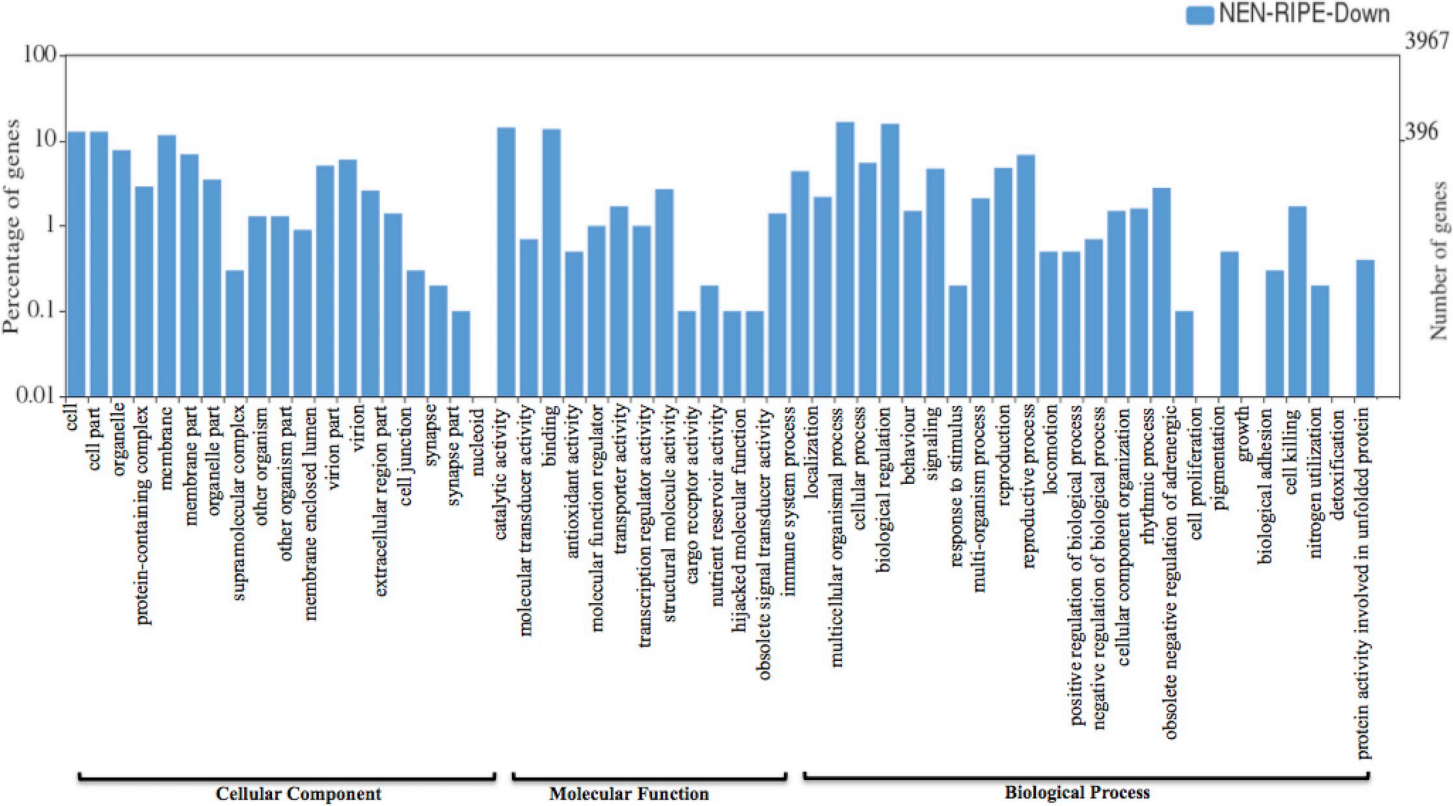
WEGO plot representing annotation and classification of differentially expressed genes in Nendran unripe fruit-pulp. X-axis (right) displaying selected GO terms of down-regulated genes and y-axis (left) displaying the percentage of genes.

The significantly differentially expressed genes were then mapped to the banana genome hub database (https://banana-genome-hub.southgreen.fr/). Further, the study on gene ontology (GO) and classification of DEGs was performed to get the information of genes involved in cellular, molecular and biological processes in ripened fruit pulp of Nendran. All the DEGs with annotated GO terms were visualized using the WEGO tool (**Figs 2 and 3**). In the cellular component, most of the genes were classified into the extracellular region, cell part and membrane part, while in the molecular function, most of the genes were involved in catalytic activity, binding and molecular function regulators. In biological processes, genes were mainly involved in response to stimuli, biogenesis or cellular component organization, biological regulation, etc. Overall, cellular component organization, localization, developmental process and response to stimuli were the most considerably enriched processes in DEGs **(S1 Fig)**.

### Identification of differentially expressed genes

**Fig 4:**
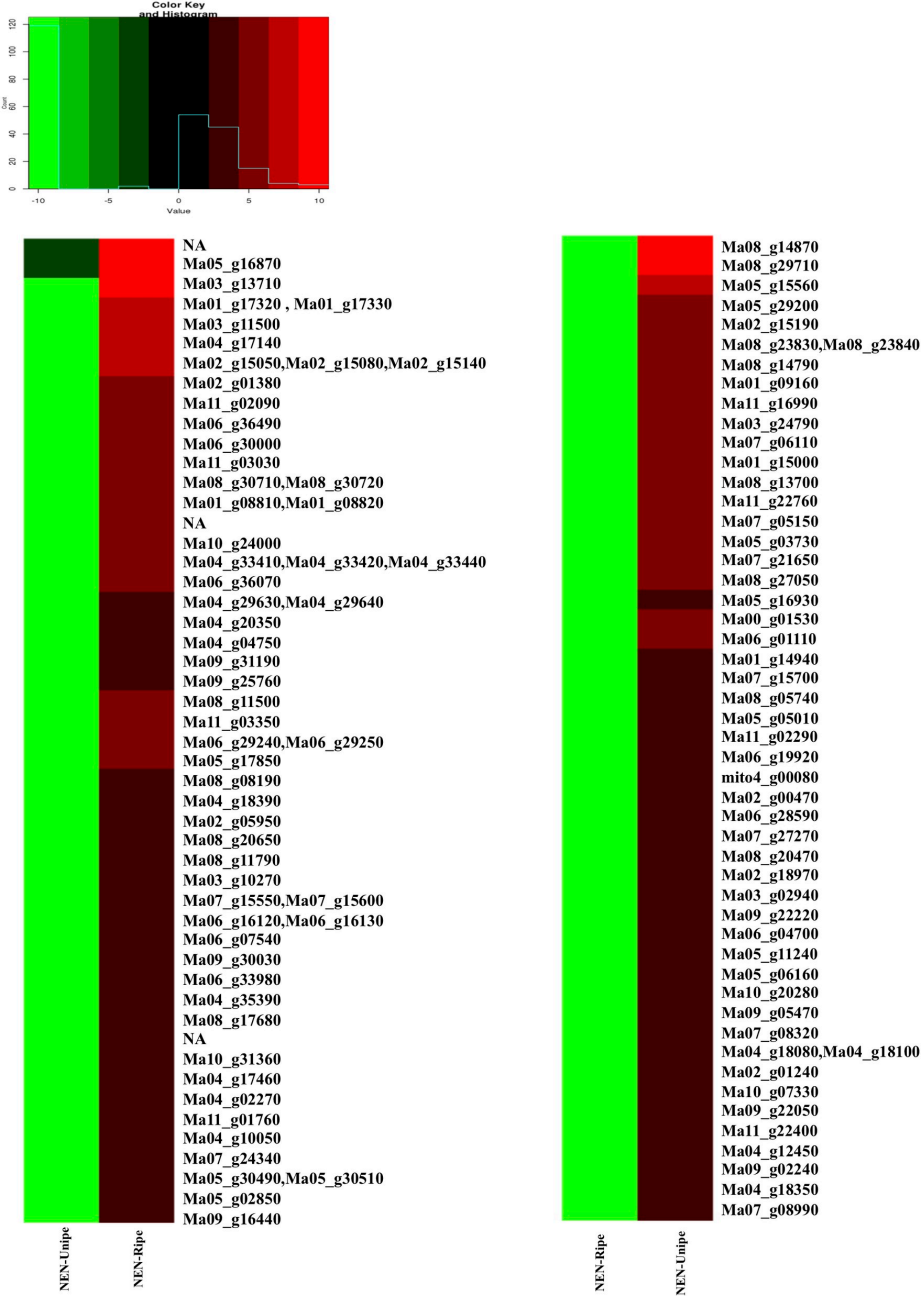
**Clustered heatmap depicting top 50 up-regulated (left panel) and down-regulated genes (right panel) in ripe fruit pulp.** Red color indicates up-regulated genes and green color indicates down-regulated genes in Nendran fruit samples.

To identify transcripts that are expressed differentially in response to ripening, the top 50 up-regulated and down-regulated genes were selected for further analysis. Gene expression of the most up-regulated transcripts varied from 14 to 6.6 folds (**Table 2**). The acyl carrier protein and cytochrome P450 encoding genes are shown to be up-regulated significantly. Categorically, other genes encoding for stress and pathogenesis-related proteins were also up-regulated in ripen fruit-pulp samples. Similarly, the genes that were down-regulated are mostly linked with TF, hydrolase, and cellulose related genes. A detailed list of top up- and down-regulated genes is depicted by a heat map (**Fig 4**). The top 50 up- and down-regulated genes are listed in **Tables 2 and 3,** respectively.

**Table 2:** List of top 50 up-regulated genes in Nendran ripe fruit samples.

*Musa acuminata* IDs	Unripe FPKM	Ripe FPKM	Log2 (fold change)	P-value
NA	0.084	1631.93	14.2455	0.00435
Ma05_g16870	0.2272	2196.7	13.2392	0.22265
Ma03_g13710	0.3616	2292.69	12.6306	0.22785
Ma01_g17320, Ma01_g17330	0.0881	368.309	12.0301	0.17305
Ma03_g11500	0.1433	473.92	11.881	0.00435
Ma04_g17140	0.3154	663.35	11.0384	0.0315
Ma02_g15050,Ma02_g15080, Ma02_g15140	0.2177	387.42	10.7974	0.04925
Ma02_g01380	0.691	1051.4	10.5714	0.03525
Ma11_g02090	0.0557	45.48	9.674	0.01545
Ma06_g36490	0	676.19	9.4034	5.00E-05
Ma06_g30000	3.1304	1572.4	8.9724	0.0368
Ma11_g03030	0.0297	13.42	8.8177	0.0439
Ma08_g30710, Ma08_g30720	2.3397	1055.65	8.8176	0.0006
Ma01_g08810, Ma01_g08820	5.11	2165.23	8.727	5.00E-05
NA	0.0754	30.64	8.6664	0.00435
Ma10_g24000	0.0244	9.9	8.666	0.04935
Ma04_g33410, Ma04_g33420, Ma04_g33440	5.7266	2293.89	8.6459	5.00E-05
Ma06_g36070	0.0738	27.39	8.5367	0.0104
Ma04_g29630, Ma04_g29640	6.4843	2380.04	8.5198	0.00375
Ma04_g20350	3.3082	1206.63	8.5107	0.00055
Ma04_g04750	21.9975	6325.5	8.1677	0.03775
Ma09_g31190	2.5786	741.18	8.1671	0.00955
Ma09_g25760	0.5324	135.71	7.9938	0.00875
Ma08_g11500	2.4939	631.55	7.9844	0.00035
Ma11_g03350	0.2043	50.78	7.9573	0.037
Ma06_g29240, Ma06_g29250	0.1632	40.08	7.9398	0.01065
Ma05_g17850	1.8005	431.87	7.9061	0.0191
Ma08_g08190	0.3605	78.63	7.7691	0.01995
Ma04_g18390	4.12	756.6	7.5207	0.00025
Ma02_g05950	3.5512	617.78	7.4427	0.0025
Ma08_g20650	0.0493	8.37	7.4071	0.01545
Ma08_g11790	0.072	11.7	7.3435	0.00455
Ma03_g10270	0	159.22	7.3239	5.00E-05
Ma07_g15550, Ma07_g15600	2.0088	300.7	7.2259	0.0034
Ma06_g16120, Ma06_g16130	2.204	318.29	7.1741	0.00385
Ma06_g07540	0.282	39.53	7.1312	0.00865
Ma09_g30030	0.4778	62.16	7.0235	0.02725
Ma06_g33980	4.2437	547.44	7.0112	0.00185
Ma04_g35390	1.1122	141.47	6.9909	0.00045
Ma08_g17680	0.2461	30.13	6.9358	0.03105
NA	0	120.5	6.9248	0.00535
Ma10_g31360	0.2208	24.87	6.8157	0.0103
Ma04_g17460	0.1326	14.71	6.7938	0.00435
Ma04_g02270	7.4929	825.12	6.7829	0.0047
Ma11_g01760	0.1312	14.42	6.7803	0.01715
Ma04_g10050	0.2748	30.15	6.7779	0.0362
Ma07_g24340	7.0938	740.83	6.7064	0.005
Ma05_g30490, Ma05_g30510	4.3015	446.84	6.6988	0.00115
Ma05_g02850	0	98.94	6.6429	0.01335
Ma09_g16440	4.0667	387.78	6.5753	0.0065

**Table 3:** List of top 50 down-regulated genes in Nendran ripe fruit samples.

*Musa acuminata* IDs	Unripe FPKM	Ripe FPKM	Log2 (fold change)	P-value
Ma08_g14870	57.9970	12.3140	-2.2357	0.0422
Ma08_g29710	18.6179	3.6973	-2.3321	0.0459
Ma05_g15560	198.1540	38.7050	-2.3560	0.04935
Ma05_g29200	82.2009	15.9326	-2.3672	0.0323
Ma02_g15190	6.4337	1.2186	-2.4005	0.04805
Ma08_g23830, Ma08_g23840	53.1387	9.9826	-2.4123	0.03
Ma08_g14790	90.0315	16.7500	-2.4263	0.04685
Ma01_g09160	2.6453	0.4839	-2.4508	0.0317
Ma11_g16990	12.0605	2.0735	-2.5402	0.04245
Ma03_g24790	11.7536	2.0114	-2.5468	0.03935
Ma07_g06110	15.2059	2.5859	-2.5559	0.0428
Ma01_g15000	16.7579	2.8328	-2.5646	0.038
Ma08_g13700	55.7171	9.4159	-2.5649	0.04005
Ma11_g22760	24.0255	4.0311	-2.5753	0.0439
Ma07_g05150	13.5948	2.2438	-2.5990	0.04545
Ma05_g03730	18.2312	2.9992	-2.6038	0.0464
Ma07_g21650	31.7084	5.1810	-2.6136	0.02875
Ma08_g27050	23.9941	3.9084	-2.6180	0.037
Ma05_g16930	23.3441	3.7838	-2.6252	0.0362
Ma00_g01530	20.2872	3.2693	-2.6335	0.0239
Ma06_g01110	30.6584	4.9257	-2.6379	0.04155
Ma01_g14940	136.9260	21.7356	-2.6553	0.03425
Ma07_g15700	179.5230	28.0934	-2.6759	0.0445
Ma08_g05740	29.6195	4.6249	-2.6791	0.04335
Ma05_g05010	105.5100	16.4617	-2.6802	0.0486
Ma11_g02290	12.5888	1.9597	-2.6835	0.0328
Ma06_g19920	10.2797	1.5929	-2.6901	0.02795
mito4_g00080	10.7582	1.6646	-2.6922	0.0327
Ma02_g00470	175.1800	27.0540	-2.6949	0.02425
Ma06_g28590	32.6655	5.0367	-2.6972	0.0341
Ma07_g27270	54.7385	8.3345	-2.7154	0.02555
Ma08_g20470	54.1184	8.2262	-2.7178	0.04435
Ma02_g18970	29.7441	4.4848	-2.7295	0.04965
Ma03_g02940	23.3713	3.5238	-2.7296	0.0315
Ma09_g22220	45.9947	6.9188	-2.7329	0.03515
Ma06_g04700	16.4684	2.4648	-2.7402	0.0328
Ma05_g11240	186.9100	27.9561	-2.7411	0.02375
Ma05_g06160	84.3011	12.5721	-2.7453	0.0389
Ma10_g20280	15.5856	2.3120	-2.7530	0.04475
Ma09_g05470	20.7883	3.0835	-2.7531	0.04635
Ma07_g08320	193.6760	28.4678	-2.7662	0.04545
Ma04_g18080, Ma04_g18100	18.5819	2.7309	-2.7665	0.01615
Ma02_g01240	460.2760	67.5250	-2.7690	0.02775
Ma10_g07330	34.0326	4.9770	-2.7736	0.02735
Ma09_g22050	38.0633	5.5625	-2.7746	0.0358
Ma11_g22400	20.6719	2.9902	-2.7894	0.03615
Ma04_g12450	95.9664	13.8414	-2.7935	0.04895
Ma09_g02240	26.2597	3.7843	-2.7947	0.03605
Ma04_g18350	20.4148	2.9223	-2.8045	0.02455

### Differential expression pattern of genes involved in the carotenoid biosynthesis pathway

The fruit ripening response and the expression of carotenoid biosynthesis pathway genes in cv. Nendran was correlated and presented in **S2 Fig.** It was observed that expression of *isopentenyl-diphosphate delta isomerase 1-like (IPP*) (1 fold), *lycopene beta cyclase (LCYβ)* (1.29 fold), *geranylgeranyl pyrophosphate synthase (GGPS*) (6 fold), *prolycopene isomerase (CRTISO)* (1.5 fold), *cytokinin dehydrogenase* (5 fold), *4-hydroxy-3-methylbut-2-enyl diphosphate synthase* (*HDS*) (4 fold) and *phytoene desaturase* (*PDS*) (1.44 fold) was significantly enhanced at the ripe stage of fruit compared to the unripe stage. While the gene expression of *9-cis-epoxycarotenoid dehydrogenase (NCED)*, *carotenoid 9%2C10(9%2C10’)-cleavage dioxygenase 1-like (CCD1)*, *lycopene epsilon cyclase (LCYε)*, *β-carotene 3-hydroxylase 2* (*BCH2)* and *phytoene synthase 2 (PSY2)* was highly down-regulated at ripened stage (ranging from -1 to -9 folds) (**S2 Fig**).

### Genes involved in ripening, aroma and flavor

The banana ripening process is known to be involved in softening of fruit tissues that lead to the formation of aromatic compounds. The softening is mainly governed by the degradation of cell wall components [36]. This process is associated with a repertoire of genes, which are differentially expressed to regulate these events. Therefore, we have also analyzed the expression of genes that are linked with the ripening, flavor and aroma formation.

The expression pattern of *methyltransferase*, *expansins*, *pectin lyase (PL)*, *xyloglucan endotransglucosylase*/*hydrolase protein 32* (*XTH)*, *polygalactouronase (PG)* which are ripening associated genes has been evaluated in the transcriptome data generated at the ripe stage of Nendran. The expression of the *XTH* gene family was highest (13 fold) followed by *PL* (12 fold) and *trans-resveratrol di-O-methyltransferase-like* (11 fold) in ripe fruit-pulp tissue in comparison to the unripe stage of fruit-pulp.

From the putative *XTH* gene family, 15 genes were up-regulated with the highest fold change (13 fold) and 17 genes were down-regulated (-7 fold). Similarly, six genes from the *PL* family were highly expressed during ripening stage of the banana. Nine *expansin* genes were identified and their expression was increased up to 9 fold. Few members of the *PG* gene family were highest in expression (up to 6 fold), while other gene families like *glucosidases* (GSMUA_Achr11G06230_001) (7 fold) are also expressed in the fruit-pulp of cv. Nendran. However, the expression of some of the members of these gene families like *pectinesterase* (5 fold) was considerably enhanced but still on the lower side when compared to the expression of other ripening associated families such as *XTH*. The expression details of ripening associated genes are provided in the **S2 Table**. Genes involved in softening of the cell wall were amongst the highly up-regulated genes (*PL*, *PE*, *XTH*) indicating that softening is the main event during the ripening of banana.

The presence of various volatiles viz. butyl acetate, isoamyl alcohol, and isoamyl acetate attributes to the aroma of banana fruit. Fatty acid biosynthesis and other pathways like the phenylpropanoid pathway mainly produce these volatile compounds. In this study, the expression pattern of genes involved in the biosynthesis pathways of fatty acid, unsaturated fatty acid and amino acid formation was analyzed. We have checked the expression of *alcohol dehydrogenase* (*ADH*) that mediates the conversion of alcohol from sugars. *ADH* genes are usually expressed during the fruit ripening process and reported to play a major role in the development of flavor. In total 5 transcripts annotated for *ADH* (*ADH1—ADH5*) were identified and among them *ADH1* (GSMUA_Achr2G08040_001) has shown one-fold increased expression, while others have not indicated any significant change in expression levels. Likewise, *lipoxygenases* (*LOX*) genes are also involved in aroma development and the expression of one gene belonging to *LOX* (GSMUA_Achr9G12470_001) was up-regulated (5 fold) in ripened banana. Further analysis of transcriptome data revealed that various transferases like *benzoyltransferases*, *methyltransferases* and *acyltransferases* were significantly up-regulated in ripe banana indicating their potential role in the aroma. The highest expression was observed in putative *3-N-debenzoyl-2-deoxytaxol N-benzoyltransferase* that was increased maximum by 12 fold in a ripe banana. Similarly, *3-oxoacyl-[acyl-carrier-protein] reductase* and *acyltransferase* genes also exhibited 11 and 2 fold increased expression, respectively (**S3 Table**).

Ethylene exposure also accelerates ripening in banana. ACC synthase (ACS) and ACC oxidase (ACO) are the main regulators that govern ethylene biosynthesis in fruits [37]. ACO is also considered to be the rate-limiting step in ethylene production [38]. In the current study, expression change in *ACS* and *ACO* genes to understand their role in fruit ripening of cv. Nendran is explored. It has been observed that the expression of *ACS* (3 fold) and *ACO* (1 fold) was up-regulated in a ripe fruit-pulp than that of unripe fruit-pulp (**S4 Table**).

### Identification of Transcription Factors (TFs)

**Fig 5:**
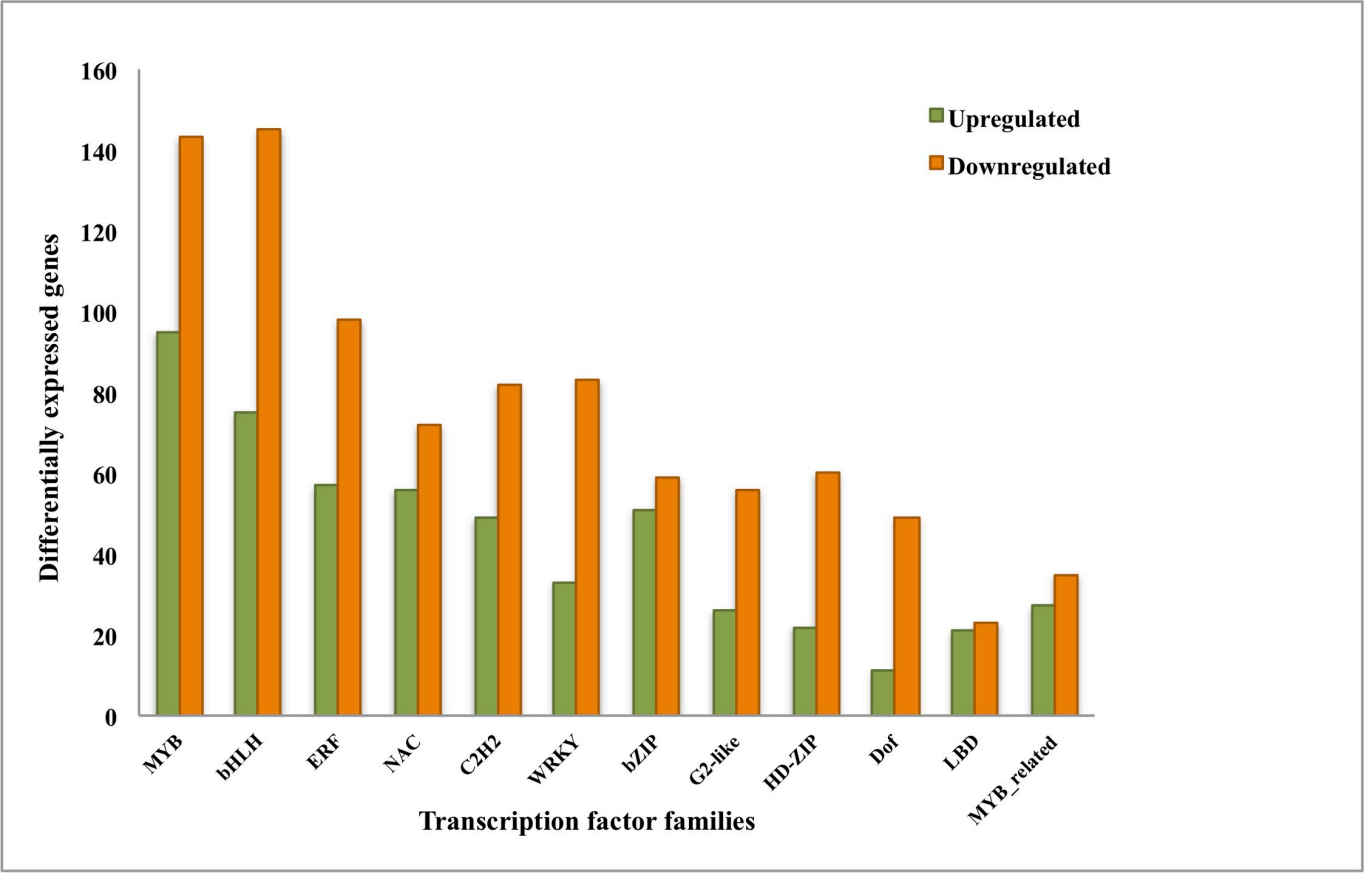
Transcription factors expressively correlated with cv. Nendran ripe fruit-pulp. Orange bars represent down-regulated genes while green bars represent up-regulated genes.

TFs regulate the expression of genes. Hence, we have downloaded TFs from the PlantTFDB database [39]. This database harbors 2896 TFs from *Musa acuminata* which are classified into 57 families. It was examined that most of the TFs belong to the multigene family and mainly the TFs were related to *MYB*, *bHLH*, *ERF*, *NAC* and *C2H2* gene family. These gene families showed varied expression at the ripening stage (up and down). A list of the top 12 TF family members is given in **Fig 5** and a detailed list of all transcription factors family members along with fold change expression is provided in the **S5 Table.**

### Validation of differential gene expression by qRT-PCR

**Fig 6:**
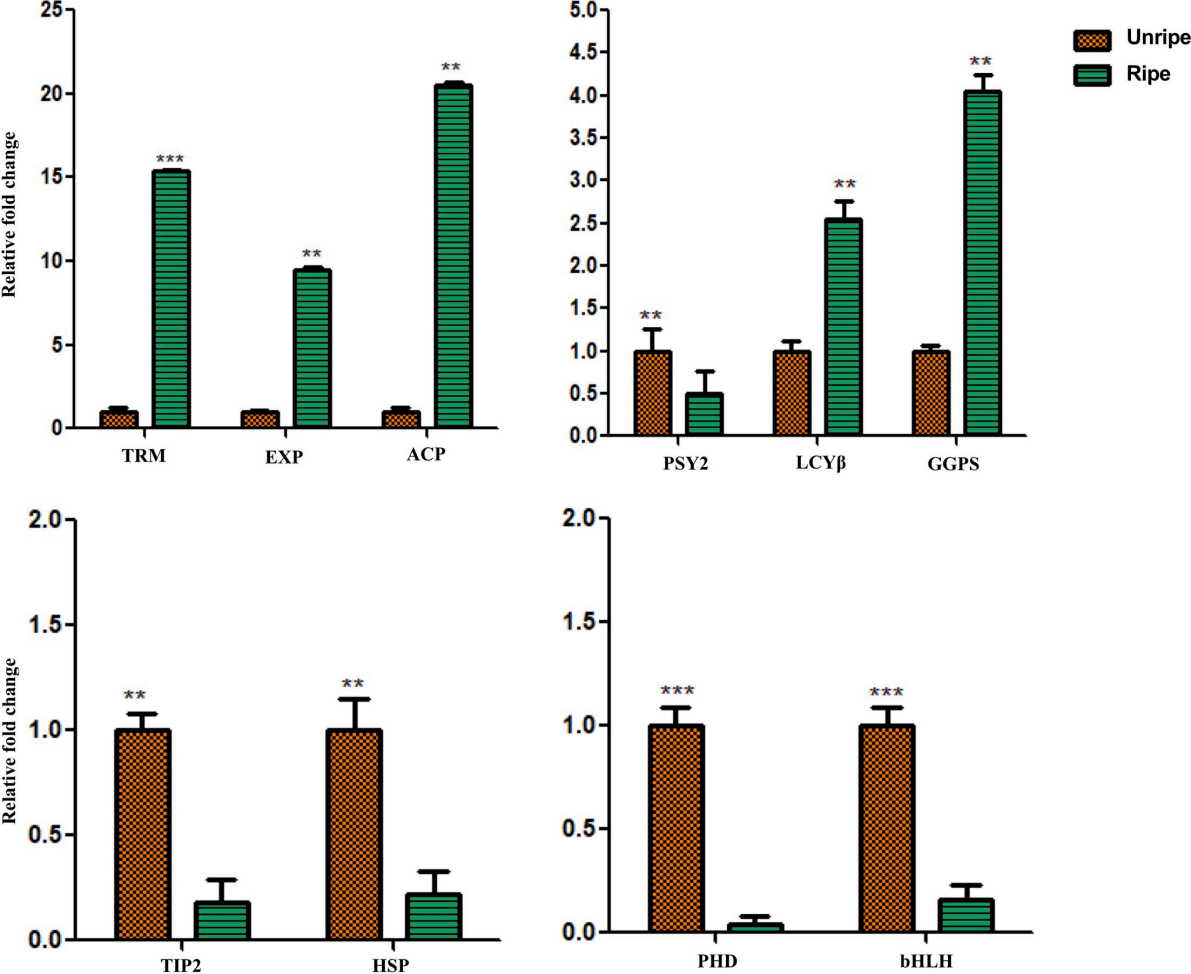
Quantitative real-time PCR of selected genes. Expression profile of selected genes in ripe Nendran banana. Gene expression was normalized using *Actin1* as an internal control. Orange bars represent the expression of unripe while green bars depict the expression of ripe genes. Statistical analysis was executed using Student’s paired t-test and statistical significance was checked at **P ≤ 0.001; ***P ≤ 0.0001. *TRM (Trans-resveratrol di-O-methyltransferase); EXP (Expansin-A2); ACP (Putative 3-oxoacyl-[acyl-carrier-protein] reductase*, *chloroplastic); PSY2 (phytoene synthase 2); LCYβ (lycopene beta cyclase); GGPS (geranylgeranyl pyrophosphate synthase); TIP2 (*Probable aquaporin *TIP2);* HSP (Heat shock 70 kDa protein 8)*; PHD* (PHD-type domain-containing protein)*; bHLH (transcription factor bHLH62)*.(Corresponding gene IDs given in **S1 Table**).

The validation of differential expression of selected genes belonging to different pathways was checked by qRT-PCR assay. Total ten genes were selected based on their significant differential expression pattern and potential role in acting as TFs stress response and carotenoid pathway genes. All the genes exhibited a comparable trend of expression in unripe and ripe stages as attained by transcriptomic data. It was observed that expression of *putative 3-oxoacyl-[acyl-carrier-protein] reductase*, *chloroplastic* (*ACP*), *trans-resveratrol di-O-methyltransferase* (*TRM*) and *expansin-A2* (*EXP*) was up-regulated which is in accordance with the transcriptome data. Similarly, the expression of genes involved in the carotenoid pathway *LCYβ* and *GGPS* was highly up-regulated in ripe fruit-pulp of cv. Nendran as compared to unripe sample (**Fig 6**). Further, the expression of *Actin 1* (LOC103992183) was also analysed and no change in expression pattern was observed in both ripe and unripe conditions.

## Discussion

Banana is a staple fruit crop worldwide. Therefore, the understanding of molecular mechanisms that are associated with various traits is crucial. Reports showed that ripening in various fruit crops including banana is initiated by a set of genes that brings physico-chemical changes in the quality of fruit [40]. These changes mainly alter cell wall degradation, synthesis of volatile compounds and alteration in phenolic constituents [40]. Sequencing technologies now provide ways to identify ripening and associated process-related genes and subsequently those can be used for genetic manipulation for the improvement of banana. Henceforth, insights into the genes responsible for ripening are necessary to understand the molecular basis of these genes that can also be implemented to improve post-harvest losses of this highly perishable crop.

In the present study, cv. Nendran of banana has been selected as it is having a high content of carotenoid at the ripening stage of fruit-pulp [12]. Further, early ripening (15w of bunch emergence) of fruits in this nutritional rich cultivar has also influenced us to understand the molecular basis of this observation. Using transcriptome analysis hosted on Illumina (HiSeq 2500) 3474 up- and 4727 down-regulated genes were obtained in the Nendran ripe fruit-pulp samples. Functional enrichment of the genes revealed their probable role in various metabolic processes.

Nendran has been reported as an excellent source of β-carotene and its activity was correlated with enhanced antioxidant activity [12]. Ripening plays a crucial role in the biosynthesis of carotenoids in the fruit-pulp tissue of banana [41]. In the current study, the expression pattern of carotenoid genes indicated that *LCYβ*, *GGPS*, and *PDS* were up-regulated in ripe fruit pulp. Moreover, the role of *LCYβ* in β-carotene biosynthesis has already been demonstrated by the regulation of lycopene flux [42]. Similarly, *GGPS* serves as a precursor for important compounds like tocopherols, carotenoids, and chlorophyll [43]. Expression of *GGPS* was up-regulated in ripe pulp samples. *PDS* is one of the first enzymes in the carotenoid biosynthesis pathway. It has also been reported as a positive regulator for ripening in tomato fruit [44]. Mutation in *PDS* gene by CRISPR/Cas technology resulted in decreased chlorophyll and carotenoid content in banana cv. Rasthali [32]. The transcriptome data have shown a ~2 fold increase in expression of the *PDS* gene (GSMUA_Achr3G21400_001) in the ripe fruit. It indicates the possible role of *PDS* in early fruit ripening and high carotenoid deposition in cv. Nendran. *BCH* gene is reported to involve in the biosynthesis of zeaxanthin which is a precursor of abscisic acid [45]. In our analysis expression of gene annotated as *β-carotene 3-hydroxylase* 2 (*BCH2*) (GSMUA_Achr11G02930_001) in *Musa* was down-regulated by ~ -6 fold. Other carotenoid pathway genes like *CCD*, *NCED* are considered carotenoid degrading enzymes [46]. In this study expression of these genes was down-regulated in ripened banana, signifying their less role in the ripening and carotenoid degradation process in Nendran.

Softening is mainly initiated with the inception of ripening [47], which leads to cell wall degradation. Cell wall hydrolysis plays a crucial role in plant growth and development, stress response and ripening process. Utmost of the genes involved in this process are mainly members of multigene families and are associated with specified functions like cell wall metabolism [48]. The significant cell wall degrading proteins are *pectin methyl esterase*, *polygalacturonase (PG)*, *XTH*, *expansins*, *PL*, galactosidases and endoglucanases [49]. *XTH* gene family members have been reported to play important role in the ripening of fruits like tomato and apple [50, 51]. Studies in fruit crops such as mango and banana have reported the role of these genes in cell wall loosening [52, 53]. The expression pattern of most of the genes belonging to *XTH* and *expansin*s gene families was reported to be highly up-regulated during ripening conditions. Amongst them, some members of these gene families were also down-regulated indicating that they might not be playing any significant function in the ripening process.

Ethylene is considered one of the major plant hormones that control many aspects of fruit ripening [54]. The initial step in ethylene biosynthesis is the conversion of S-adenosyl methionine to 1-aminocyclopropane-1-carboxylic-acid catalyzed by ACS [55]. The *ACS* and *ACO* genes are reported to regulate ethylene biosynthesis in the tomato and apple during the fruit ripening stage [56, 57]. Transcriptome data in this study revealed up-regulation of both genes in ripe fruit-pulp tissue specifying their potential role in ethylene synthesis and ripening.

We also analysed the expression of transcription gene families in fruit-pulp of cv. Nendran and found that most of the members of these multigene families of TFs were down-regulated at the ripening stage. These TFs may not be required at this stage hence, their expression is declined during the ripening process.

## Conclusion

The comparative analysis of transcriptome at the unripe and ripen stages of fruit-pulp of cv. Nendran provides a comprehensive landscape of differentially expressed genes that are mostly associated with ripening, carotenoid biosynthesis, aroma and other related processes. The expression data acquired by RNA-seq were validated by qRT-PCR analysis. The results of this study suggested that many differentially expressed genes in the unripe and ripe banana are associated with aroma and ripening processes. Gene families in ripening like *PL*, *expansins*, *XTH* etc. were showed differential expression patterns. Genes like *acyltransferases* known to be responsible for cell wall hydrolysis and production of aromatic volatiles and flavor have shown higher expression at the ripening stage. The expression pattern of the carotenoid synthesis pathway genes indicated that *LCYβ* and *GGPS* were highly up-regulated during the ripening stage while *CCDs* and *NCEDs* were downregulated. Overall, the present study has provided information about the promising role of genes such as *acyltransferases*, *LCYβ* and *GGPS* to develop a better understanding of the ripening process and their link with carotenoid synthesis, aroma and flavor formation in banana fruit-pulp.

## Supporting information

S1 Fig List of enriched GO terms in differentially expressed genes in fruit-pulp of Nendran. X-axis represents significance of gene ontology term enrichment and y-axis represents the log P-values.
(PDF)

S2 Fig Heatmap of differentially expressed genes involved in carotenoid biosynthesis pathway. Red color represents up-regulated genes and green color represents down-regulated genes.
(PDF)

S1 Table Primers used in quantitative real-time PCR study. (XLSX)

S2 Table Expression pattern (log2 fold change) of the genes involved in ripening. (XLSX)

S3 Table Expression pattern (log2 fold change) of the genes involved in aroma and flavor. (XLSX)

S4 Table Expression pattern (log2 fold change) of the genes involved in ethylene synthesis. (XLSX)

S5 Table List of Transcription Factors (TFs) family members with the expression in log2 fold change. (XLSX)

## References

[1] Heslop-Harrison JS, Schwarzacher T. Domestication, genomics and the future for banana. Annals of botany. 2007;100(5):1073–84. doi: 10.1093/aob/mcm191 17766312 PMC2759213

[2] Wang Z, Miao H, Liu J, Xu B, Yao X, Xu C, et al. Musa balbisiana genome reveals subgenome evolution and functional divergence. Nature plants. 2019;5(8):810–21. doi: 10.1038/s41477-019-0452-6 31308504 PMC6784884

[3] de Jesus ON, Silva Sde O, Amorim EP, Ferreira CF, de Campos JM, Silva Gde G, et al. Genetic diversity and population structure of Musa accessions in ex situ conservation. BMC plant biology. 2013;13:41. doi: 10.1186/1471-2229-13-41 23497122 PMC3636076

[4] Manning K, Tör M, Poole M, Hong Y, Thompson AJ, King GJ, et al. A naturally occurring epigenetic mutation in a gene encoding an SBP-box transcription factor inhibits tomato fruit ripening. Nature genetics. 2006;38(8):948–52. doi: 10.1038/ng1841 16832354

[5] Clendennen SK, May GD. Differential gene expression in ripening banana fruit. Plant physiology. 1997;115(2):463–9. doi: 10.1104/pp.115.2.463 9342866 PMC158503

[6] Sanchita Biswas Murmu HNM. Post-harvest shelf-life of banana and guava: Mechanisms of common degradation problems and emerging counteracting strategies. Innovative Food Science & Emerging Technologies. 20 July 2018;49:20–30.

[7] Maqbool M AA, Ramachandran S, Smith DR, Alderson PG. Control of postharvest anthracnose of banana using a new edible composite coating. International Society for Horticultural Science. 2010:639–44.

[8] Elitzur T, Vrebalov J, Giovannoni JJ, Goldschmidt EE, Friedman H. The regulation of MADS-box gene expression during ripening of banana and their regulatory interaction with ethylene. Journal of experimental botany. 2010;61(5):1523–35. doi: 10.1093/jxb/erq017 20200120 PMC2837265

[9] Adams-Phillips L, Barry C, Giovannoni J. Signal transduction systems regulating fruit ripening. Trends in plant science. 2004;9(7):331–8. doi: 10.1016/j.tplants.2004.05.004 15231278

[10] Yan SC, Chen JY, Yu WM, Kuang JF, Chen WX, Li XP, et al. Expression of genes associated with ethylene-signalling pathway in harvested banana fruit in response to temperature and 1-MCP treatment. Journal of the science of food and agriculture. 2011;91(4):650–7. doi: 10.1002/jsfa.4226 21302318

[11] Englberger L, Wills RB, Blades B, Dufficy L, Daniells JW, Coyne T. Carotenoid content and flesh color of selected banana cultivars growing in Australia. Food and nutrition bulletin. 2006;27(4):281–91. doi: 10.1177/156482650602700401 17209469

[12] Kaur N, Pandey A, Shivani, Kumar P, Pandey P, Kesarwani AK, et al. Regulation of Banana Phytoene Synthase (MaPSY) Expression, Characterization and Their Modulation under Various Abiotic Stress Conditions. Frontiers in plant science. 2017;8:462. doi: 10.3389/fpls.2017.00462 28421096 PMC5377061

[13] D’Hont A, Denoeud F, Aury JM, Baurens FC, Carreel F, Garsmeur O, et al. The banana (Musa acuminata) genome and the evolution of monocotyledonous plants. Nature. 2012;488(7410):213–7. doi: 10.1038/nature11241 22801500

[14] Wang Z, Gerstein M, Snyder M. RNA-Seq: a revolutionary tool for transcriptomics. Nature reviews Genetics. 2009;10(1):57–63. doi: 10.1038/nrg2484 19015660 PMC2949280

[15] Hrdlickova R, Toloue M, Tian B. RNA-Seq methods for transcriptome analysis. Wiley interdisciplinary reviews RNA. 2017;8(1). doi: 10.1002/wrna.1364 27198714 PMC5717752

[16] Asif MH, Lakhwani D, Pathak S, Gupta P, Bag SK, Nath P, et al. Transcriptome analysis of ripe and unripe fruit tissue of banana identifies major metabolic networks involved in fruit ripening process. BMC plant biology. 2014;14:316. doi: 10.1186/s12870-014-0316-1 25442405 PMC4263013

[17] Sun J, Zhang J, Fang H, Peng L, Wei S, Li C, et al. Comparative transcriptome analysis reveals resistance-related genes and pathways in Musa acuminata banana ’Guijiao 9’ in response to Fusarium wilt. Plant physiology and biochemistry: PPB. 2019;141:83–94. doi: 10.1016/j.plaphy.2019.05.022 31136934

[18] Nakano M, McCormick K, Demirci C, Demirci F, Gurazada SGR, Ramachandruni D, et al. Next-Generation Sequence Databases: RNA and Genomic Informatics Resources for Plants. Plant physiology. 2020;182(1):136–46. doi: 10.1104/pp.19.00957 31690707 PMC6945852

[19] Zhang A, Zhang Q, Li J, Gong H, Fan X, Yang Y, et al. Transcriptome co-expression network analysis identifies key genes and regulators of ripening kiwifruit ester biosynthesis. BMC plant biology. 2020;20(1):103. doi: 10.1186/s12870-020-2314-9 32138665 PMC7059668

[20] Qi X, Ogden EL, Die JV, Ehlenfeldt MK, Polashock JJ, Darwish O, et al. Transcriptome analysis identifies genes related to the waxy coating on blueberry fruit in two northern-adapted rabbiteye breeding populations. BMC plant biology. 2019;19(1):460. doi: 10.1186/s12870-019-2073-7 31711416 PMC6844065

[21] Blanca J, Esteras C, Ziarsolo P, Pérez D, Fernã Ndez-Pedrosa V, Collado C, et al. Transcriptome sequencing for SNP discovery across Cucumis melo. BMC genomics. 2012;13:280. doi: 10.1186/1471-2164-13-280 22726804 PMC3473316

[22] Yu K, Xu Q, Da X, Guo F, Ding Y, Deng X. Transcriptome changes during fruit development and ripening of sweet orange (Citrus sinensis). BMC genomics. 2012;13:10. doi: 10.1186/1471-2164-13-10 22230690 PMC3267696

[23] Wang JH, Liu JJ, Chen KL, Li HW, He J, Guan B, et al. Comparative transcriptome and proteome profiling of two Citrus sinensis cultivars during fruit development and ripening. BMC genomics. 2017;18(1):984. doi: 10.1186/s12864-017-4366-2 29268697 PMC5740884

[24] Sun Y, Fan M, He Y. Transcriptome Analysis of Watermelon Leaves Reveals Candidate Genes Responsive to Cucumber green mottle mosaic virus Infection. International journal of molecular sciences. 2019;20(3). doi: 10.3390/ijms20030610 30708960 PMC6387395

[25] Zhan Y, Qu Y, Zhu L, Shen C, Feng X, Yu C. Transcriptome analysis of tomato (Solanum lycopersicum L.) shoots reveals a crosstalk between auxin and strigolactone. PloS one. 2018;13(7):e0201124. doi: 10.1371/journal.pone.0201124 30044859 PMC6059464

[26] Bolger AM, Lohse M, Usadel B. Trimmomatic: a flexible trimmer for Illumina sequence data. Bioinformatics (Oxford, England). 2014;30(15):2114–20. doi: 10.1093/bioinformatics/btu170 24695404 PMC4103590

[27] Droc G, Larivière D, Guignon V, Yahiaoui N, This D, Garsmeur O, et al. The banana genome hub. Database: the journal of biological databases and curation. 2013;2013:bat035. doi: 10.1093/database/bat035 23707967 PMC3662865

[28] Dobin A, Davis CA, Schlesinger F, Drenkow J, Zaleski C, Jha S, et al. STAR: ultrafast universal RNA-seq aligner. Bioinformatics (Oxford, England). 2013;29(1):15–21. doi: 10.1093/bioinformatics/bts635 23104886 PMC3530905

[29] Trapnell C, Roberts A, Goff L, Pertea G, Kim D, Kelley DR, et al. Differential gene and transcript expression analysis of RNA-seq experiments with TopHat and Cufflinks. Nature protocols. 2012;7(3):562–78. doi: 10.1038/nprot.2012.016 22383036 PMC3334321

[30] Raudvere U, Kolberg L, Kuzmin I, Arak T, Adler P, Peterson H, et al. g:Profiler: a web server for functional enrichment analysis and conversions of gene lists (2019 update). Nucleic acids research. 2019;47(W1):W191–w8. doi: 10.1093/nar/gkz369 31066453 PMC6602461

[31] Ye J, Zhang Y, Cui H, Liu J, Wu Y, Cheng Y, et al. WEGO 2.0: a web tool for analyzing and plotting GO annotations, 2018 update. Nucleic acids research. 2018;46(W1):W71–w5. doi: 10.1093/nar/gky400 29788377 PMC6030983

[32] Kaur N, Alok A, Shivani, Kaur N, Pandey P, Awasthi P, et al. CRISPR/Cas9-mediated efficient editing in phytoene desaturase (PDS) demonstrates precise manipulation in banana cv. Rasthali genome. Functional & integrative genomics. 2018;18(1):89–99. doi: 10.1007/s10142-017-0577-5 29188477

[33] Kaur N, Alok A, Shivani, Kumar P, Kaur N, Awasthi P, et al. CRISPR/Cas9 directed editing of lycopene epsilon-cyclase modulates metabolic flux for β-carotene biosynthesis in banana fruit. Metabolic engineering. 2020;59:76–86. doi: 10.1016/j.ymben.2020.01.008 32006663

[34] Schmittgen TD, Livak KJ. Analyzing real-time PCR data by the comparative C(T) method. Nature protocols. 2008;3(6):1101–8. doi: 10.1038/nprot.2008.73 18546601

[35] Untergasser A, Cutcutache I, Koressaar T, Ye J, Faircloth BC, Remm M, et al. Primer3—new capabilities and interfaces. Nucleic acids research. 2012;40(15):e115. doi: 10.1093/nar/gks596 22730293 PMC3424584

[36] Xiao C, Anderson CT. Roles of pectin in biomass yield and processing for biofuels. Frontiers in plant science. 2013;4:67. doi: 10.3389/fpls.2013.00067 23543255 PMC3608898

[37] Bleecker AB, Schaller GE. The Mechanism of Ethylene Perception. Plant physiology. 1996;111(3):653–60. doi: 10.1104/pp.111.3.653 12226320 PMC157880

[38] Van de Poel B, Bulens I, Markoula A, Hertog ML, Dreesen R, Wirtz M, et al. Targeted systems biology profiling of tomato fruit reveals coordination of the Yang cycle and a distinct regulation of ethylene biosynthesis during postclimacteric ripening. Plant physiology. 2012;160(3):1498–514. doi: 10.1104/pp.112.206086 22977280 PMC3490579

[39] Jin J, Zhang H, Kong L, Gao G, Luo J. PlantTFDB 3.0: a portal for the functional and evolutionary study of plant transcription factors. Nucleic acids research. 2014;42(Database issue):D1182–7. doi: 10.1093/nar/gkt1016 24174544 PMC3965000

[40] Kulkarni SG, Kudachikar VB, Keshava Prakash MN. Studies on physico-chemical changes during artificial ripening of banana (Musa sp) variety ’Robusta’. Journal of food science and technology. 2011;48(6):730–4. doi: 10.1007/s13197-010-0133-y 23572812 PMC3551045

[41] Su L, Diretto G, Purgatto E, Danoun S, Zouine M, Li Z, et al. Carotenoid accumulation during tomato fruit ripening is modulated by the auxin-ethylene balance. BMC plant biology. 2015;15:114. doi: 10.1186/s12870-015-0495-4 25953041 PMC4424491

[42] Zeng J, Wang C, Chen X, Zang M, Yuan C, Wang X, et al. The lycopene β-cyclase plays a significant role in provitamin A biosynthesis in wheat endosperm. BMC plant biology. 2015;15:112. doi: 10.1186/s12870-015-0514-5 25943989 PMC4433027

[43] Beck G, Coman D, Herren E, Ruiz-Sola MA, Rodríguez-Concepción M, Gruissem W, et al. Characterization of the GGPP synthase gene family in Arabidopsis thaliana. Plant molecular biology. 2013;82(4–5):393–416. doi: 10.1007/s11103-013-0070-z 23729351

[44] Naing AH, Kyu SY, Pe PPW, Park KI, Lee JM, Lim KB, et al. Silencing of the phytoene desaturase (PDS) gene affects the expression of fruit-ripening genes in tomatoes. Plant methods. 2019;15:110. doi: 10.1186/s13007-019-0491-z 31592162 PMC6777038

[45] Du H, Wang N, Cui F, Li X, Xiao J, Xiong L. Characterization of the beta-carotene hydroxylase gene DSM2 conferring drought and oxidative stress resistance by increasing xanthophylls and abscisic acid synthesis in rice. Plant physiology. 2010;154(3):1304–18. doi: 10.1104/pp.110.163741 20852032 PMC2971608

[46] Vallabhaneni R, Bradbury LM, Wurtzel ET. The carotenoid dioxygenase gene family in maize, sorghum, and rice. Archives of biochemistry and biophysics. 2010;504(1):104–11. doi: 10.1016/j.abb.2010.07.019 20670614 PMC2957549

[47] Payasi A, Mishra NN, Chaves AL, Singh R. Biochemistry of fruit softening: an overview. Physiology and molecular biology of plants: an international journal of functional plant biology. 2009;15(2):103–13. doi: 10.1007/s12298-009-0012-z 23572919 PMC3550369

[48] Brummell DA, Harpster MH. Cell wall metabolism in fruit softening and quality and its manipulation in transgenic plants. Plant molecular biology. 2001;47(1–2):311–40. 11554479

[49] Paull RE, Chen NJ. Postharvest Variation in Cell Wall-Degrading Enzymes of Papaya (Carica papaya L.) during Fruit Ripening. Plant physiology. 1983;72(2):382–5. doi: 10.1104/pp.72.2.382 16663010 PMC1066241

[50] Saladié M, Rose JK, Cosgrove DJ, Catalá C. Characterization of a new xyloglucan endotransglucosylase/hydrolase (XTH) from ripening tomato fruit and implications for the diverse modes of enzymic action. The Plant journal: for cell and molecular biology. 2006;47(2):282–95. doi: 10.1111/j.1365-313X.2006.02784.x 16774648

[51] Muñoz-Bertomeu J, Miedes E, Lorences EP. Expression of xyloglucan endotransglucosylase/hydrolase (XTH) genes and XET activity in ethylene treated apple and tomato fruits. Journal of plant physiology. 2013;170(13):1194–201. doi: 10.1016/j.jplph.2013.03.015 23628624

[52] Srivastava S, Singh RK, Pathak G, Goel R, Asif MH, Sane AP, et al. Comparative transcriptome analysis of unripe and mid-ripe fruit of Mangifera indica (var. "Dashehari") unravels ripening associated genes. Scientific reports. 2016;6:32557. doi: 10.1038/srep32557 27586495 PMC5009307

[53] Asif MH, Nath P. Expression of multiple forms of polygalacturonase gene during ripening in banana fruit. Plant physiology and biochemistry: PPB. 2005;43(2):177–84. doi: 10.1016/j.plaphy.2005.01.011 15820666

[54] Liu M, Pirrello J, Chervin C, Roustan JP, Bouzayen M. Ethylene Control of Fruit Ripening: Revisiting the Complex Network of Transcriptional Regulation. Plant physiology. 2015;169(4):2380–90. doi: 10.1104/pp.15.01361 26511917 PMC4677914

[55] Iqbal N, Khan NA, Ferrante A, Trivellini A, Francini A, Khan MIR. Ethylene Role in Plant Growth, Development and Senescence: Interaction with Other Phytohormones. Frontiers in plant science. 2017;8:475. doi: 10.3389/fpls.2017.00475 28421102 PMC5378820

[56] Nakatsuka A, Murachi S, Okunishi H, Shiomi S, Nakano R, Kubo Y, et al. Differential expression and internal feedback regulation of 1-aminocyclopropane-1-carboxylate synthase, 1-aminocyclopropane-1-carboxylate oxidase, and ethylene receptor genes in tomato fruit during development and ripening. Plant physiology. 1998;118(4):1295–305. doi: 10.1104/pp.118.4.1295 9847103 PMC34745

[57] Tong Li DT, Xuyuan Yang, Aide Wang. Exploring the apple genome reveals six ACC synthase genes expressed during fruit ripening Author links open overlay panel. Scientia Horticulturae. 2013;157:119–23.

