# Supplementary figures and images for: Comparative transcriptome analysis of unripe and ripe banana (cv. Nendran) unraveling genes involved in ripening and other related processes
Source: PLoS One. 2021 Jul 27;16(7):e0254709. doi: 10.1371/journal.pone.0254709 (PMC8315498; doi:10.1371/journal.pone.0254709)

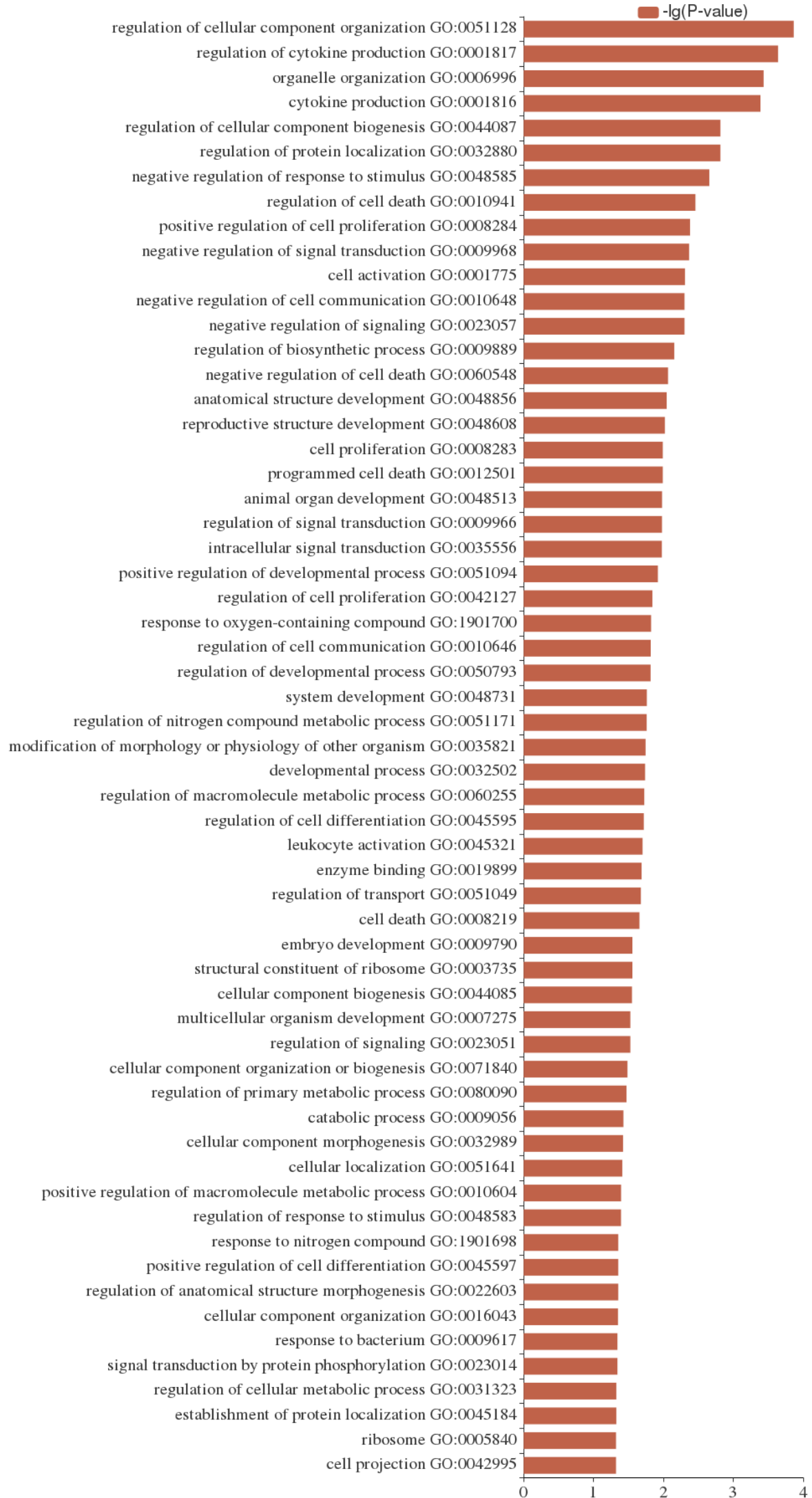

Supplement: S1 Fig — X-axis represents significance of gene ontology term enrichment and y-axis represents the log P-values. (PDF) [file pone.0254709.s001.pdf]

Color Key  
and Histogram

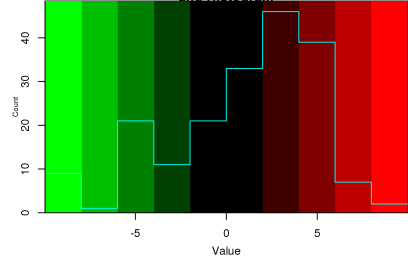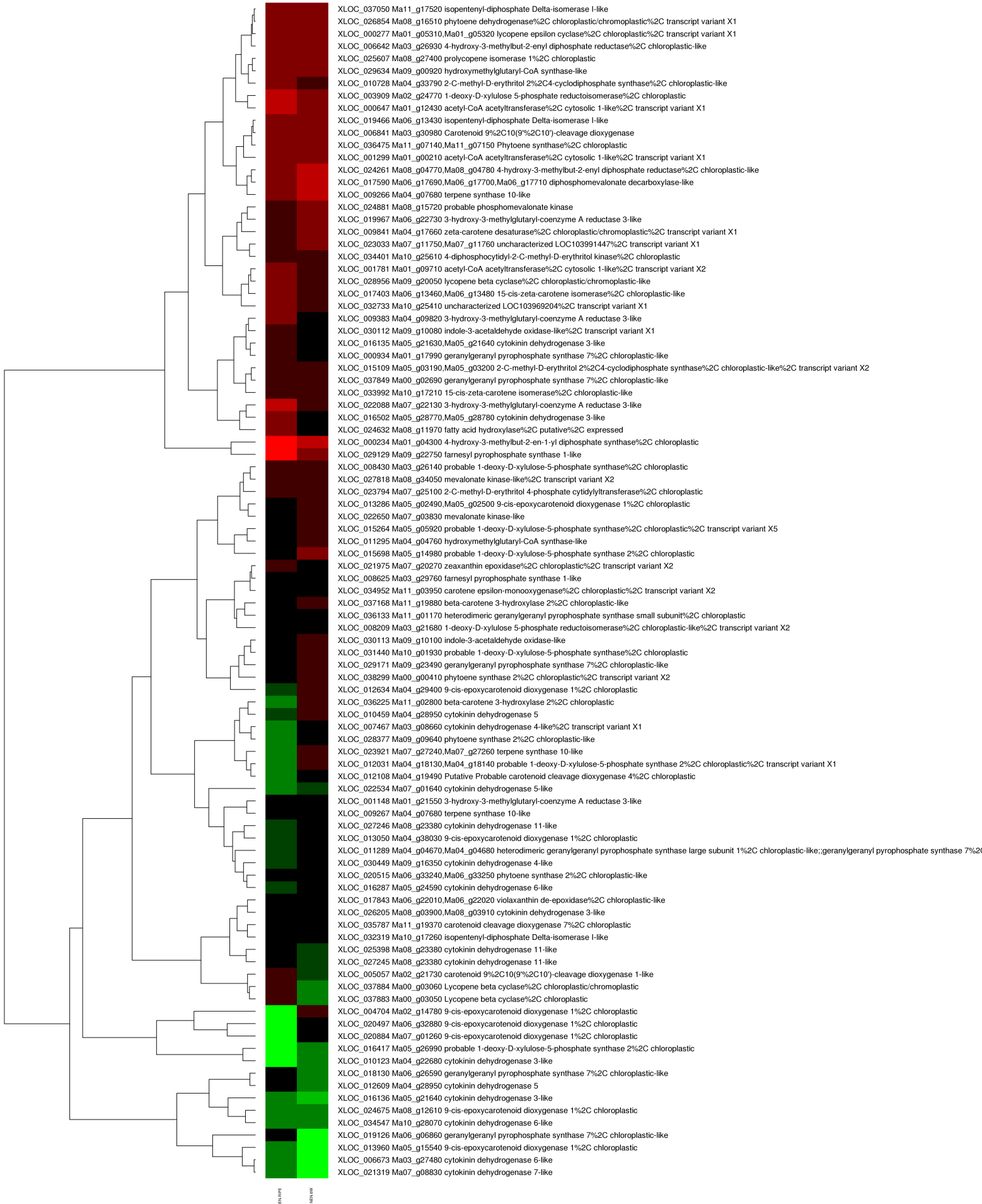

Supplement: S2 Fig — Red color represents up-regulated genes and green color represents down-regulated genes. (PDF) [file pone.0254709.s002.pdf]
